# Metabolomics in Prenatal Medicine: A Review

**DOI:** 10.3389/fmed.2021.645118

**Published:** 2021-06-25

**Authors:** Giovanni Monni, Luigi Atzori, Valentina Corda, Francesca Dessolis, Ambra Iuculano, K. Joseph Hurt, Federica Murgia

**Affiliations:** ^1^Department of Prenatal and Preimplantation Genetic Diagnosis and Fetal Therapy, Ospedale Pediatrico Microcitemico “A.Cao,” Cagliari, Italy; ^2^Clinical Metabolomics Unit, Department of Biomedical Sciences, University of Cagliari, Cagliari, Italy; ^3^Divisions of Maternal Fetal Medicine and Reproductive Sciences, Department of Obstetrics and Gynecology, University of Colorado Anschutz Medical Campus, Aurora, CO, United States

**Keywords:** congenital anatomic defects, fetal growth restriction, metabolomics, normal pregnancy, pre-eclampsia, prenatal diagnosis, prenatal medicine, pre-term labor and delivery

## Abstract

Pregnancy is a complicated and insidious state with various aspects to consider, including the well-being of the mother and child. Developing better non-invasive tests that cover a broader range of disorders with lower false-positive rates is a fundamental necessity in the prenatal medicine field, and, in this sense, the application of metabolomics could be extremely useful. Metabolomics measures and analyses the products of cellular biochemistry. As a biomarker discovery tool, the integrated holistic approach of metabolomics can yield new diagnostic or therapeutic approaches. In this review, we identify and summarize prenatal metabolomics studies and identify themes and controversies. We conducted a comprehensive search of PubMed and Google Scholar for all publications through January 2020 using combinations of the following keywords: nuclear magnetic resonance, mass spectrometry, metabolic profiling, prenatal diagnosis, pregnancy, chromosomal or aneuploidy, pre-eclampsia, fetal growth restriction, pre-term labor, and congenital defect. Metabolite detection with high throughput systems aided by advanced bioinformatics and network analysis allowed for the identification of new potential prenatal biomarkers and therapeutic targets. We took into consideration the scientific papers issued between the years 2000–2020, thus observing that the larger number of them were mainly published in the last 10 years. Initial small metabolomics studies in perinatology suggest that previously unidentified biochemical pathways and predictive biomarkers may be clinically useful. Although the scientific community is considering metabolomics with increasing attention for the study of prenatal medicine as well, more in-depth studies would be useful in order to advance toward the clinic world as the obtained results appear to be still preliminary. Employing metabolomics approaches to understand fetal and perinatal pathophysiology requires further research with larger sample sizes and rigorous testing of pilot studies using various omics and traditional hypothesis-driven experimental approaches.

## Introduction

The “-omics revolution” has brought promising options for high-throughput network-based analysis of DNA, RNA, proteins, and metabolites. Integrated analyses of the genome, transcriptome, proteome, and metabolome can reveal normal and pathophysiologic mechanisms that may not be detected by traditional hypothesis-driven experiments or focussed diagnostics (1). The analysis of end products of cellular or organismal biochemical processes using metabolomics approaches could revolutionize our approach to medical diagnosis and therapeutics. Metabolomics employs cutting-edge technologies to assess the presence of low molecular weight compounds, such as carbohydrates, amino acids, peptides, nucleic acids, organic acids, vitamins, and lipids, produced by cells, organs, or whole organisms (2, 3). The interaction and relationships among metabolites can be qualitatively and quantitatively characterized using powerful contemporary computational algorithms and increasingly effective informatics software (4). As a holistic characterization of physiology, metabolomics reflects genetics, environment, and the response to environmental stressors, and can reveal specific metabolic signatures due to genetics, disease, drugs, infection, nutrition, or exposures. Identifying metabolites or metabolic patterns that reflect specific disease states is a primary goal for metabolomics (5) research. In this review, we describe the techniques used in metabolomics, provide examples of metabolomics studies in prenatal care and maternal–fetal medicine, and highlight the tremendous opportunities for metabolomics applications in prenatal diagnosis.

### Metabolomics Overview

**Figure 1:**
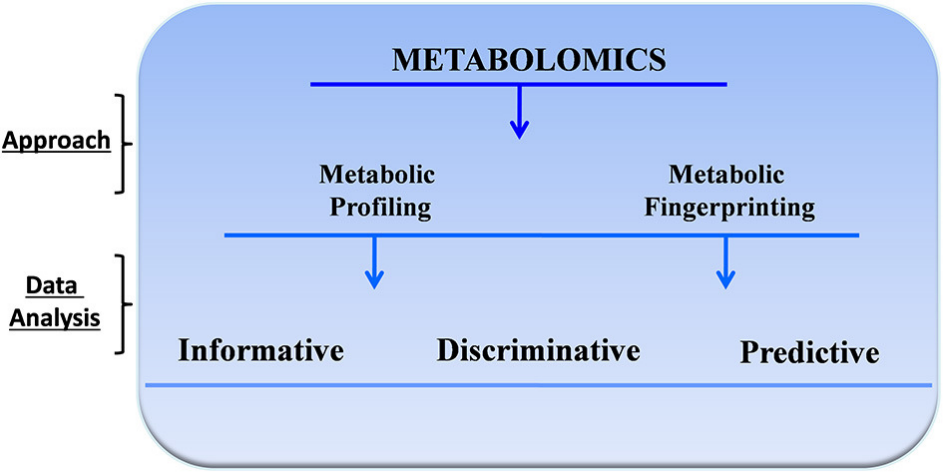
General approach for metabolomics studies.

The compounds produced by cellular metabolism are extremely diverse, involving wide variation in physicochemical properties and concentration. Their detection must be addressed by a suitable and adequate quantitative approach, and in fact, no single experimental assay can capture the full range of metabolic output (2). Therefore, investigators use multiple instruments and several analytic approaches. Two of the most common methods are metabolic profiling and metabolic fingerprinting (6). Metabolic profiling is the quantitative measurement of specific metabolites in a selected biochemical pathway or a particular class of compounds. Similarly, metabolic fingerprinting is a global screening approach that identifies metabolite patterns or “fingerprints” that are associated with known biochemical pathways, specific responses to external stimuli or endogenous signals, or disease processes. With metabolic fingerprinting, we attempt to identify discriminating metabolites that reflect specific alterations, responses, or dysfunction. Depending on the specific scientific question and the analytical approach, metabolomic analyses can be informative, discriminative, or predictive (7) (Figure 1).

Informative metabolomic analyses identify and quantify targeted or untargeted metabolites. This approach has been used to develop metabolic databases and to identify critical pathophysiologic pathways, bioactive molecules, and function or disease biomarkers. Discriminative analyses, in contrast, identify metabolic endpoint differences between samples from control and disease populations or before and after specific treatments or perturbation. This approach is typically performed using regression and other multivariate analyses to define clear diagnostic thresholds. Finally, in predictive studies, investigators use metabolic fingerprinting models based on a statistical analysis of metabolite profiles to generate predictive global algorithms that are difficult or impossible to achieve with other more focussed approaches.

Recent advances in robust high-throughput techniques such as ^1^H-Nuclear Magnetic Resonance spectroscopy (NMR) and Mass Spectrometry (MS) permit simultaneous measurement of many metabolites from a single biological sample (8–12) and more efficient metabolomic studies. Table 1 summarizes the key advantages and limitations of NMR and MS approaches in metabolomics research.

**Table 1:** The advantages and limitations of NMR spectroscopy and MS spectrometry as an analytical tool for metabolomics research.

	**Nuclear magnetic resonance**	**Mass spectrometry**
Analysis	Generally non-selective/untargeted	Both selective/targeted and non-selective/screening
Sensitivity	Lower	High using nanomolar detection limit
Reproducibility	Very high	Moderate; can depend on sample preparation or storage
Detection limits	Low micromolar to nanomolar (with specialized hardware)	Picomolar or lower (with specialized equipment)
Sample preparation	Minimal	Often requires specialized extraction, precipitation, or derivatization
Sample measurement	All metabolites detected in one measurement	Typically use different separation/preparation for different metabolite classes
Sample recovery	Non-destructive—specimen can be recovered	Destructive—but requires tiny amount of specimen
Amount of sample used	Usually 200–400 μL	2–100 μL
Number of metabolites detected in biofluids	40–200 depending on spectral resolution	>500 using various preparations
Molecular identification	Easy	Difficult
Robustness of the instruments	High	Low

**Figure 2:**
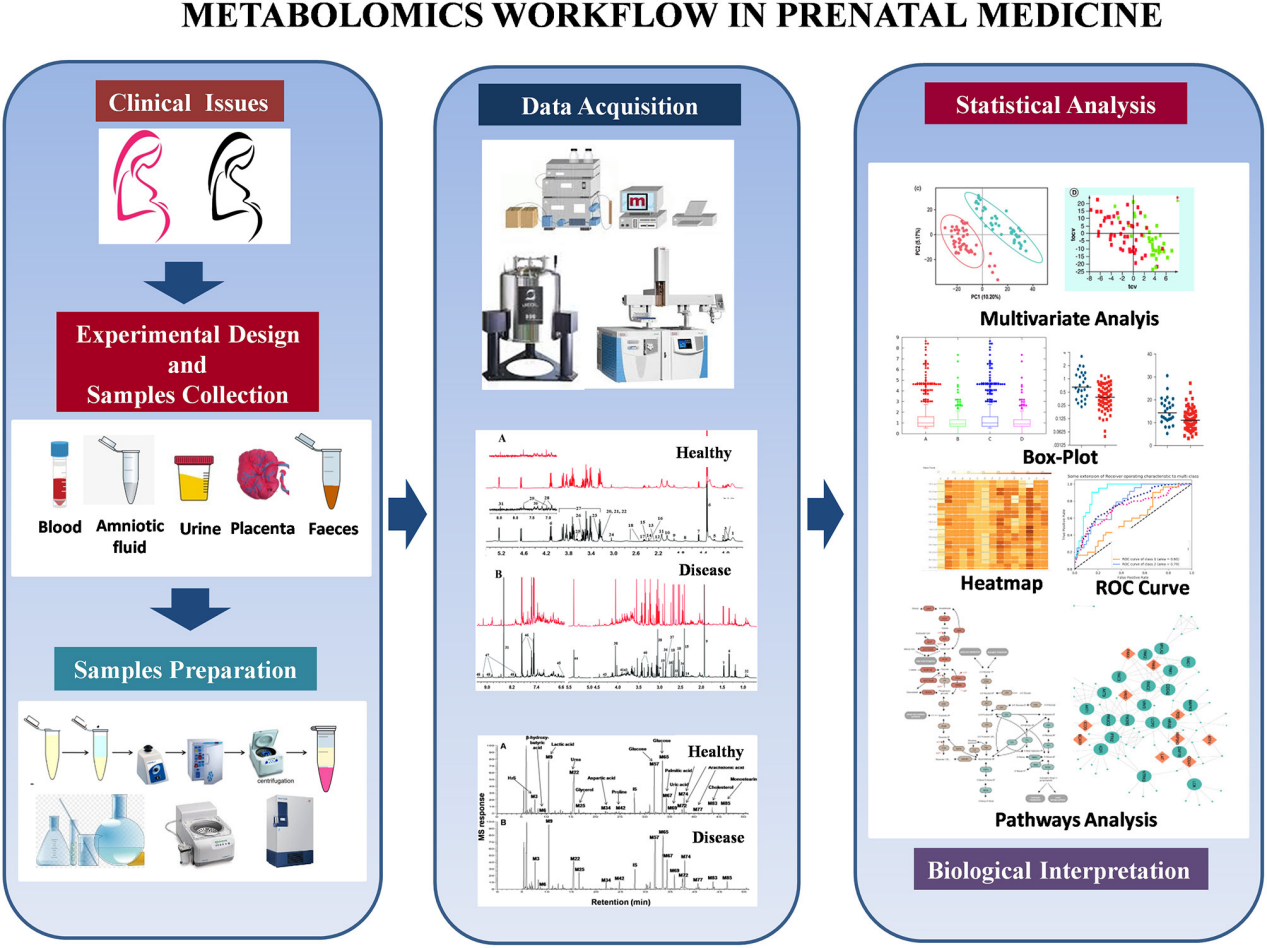
Metabolomics workflow in prenatal medicine.

Although NMR and MS are powerful techniques, the voluminous raw metabolomics data produced is only valuable after careful organization and interpretation with sophisticated contemporary bioinformatics and biostatistical tools (13, 14). Multivariate or principal component analysis transforms an assortment of metabolites into informative profiles by comparing critical elements (or components) that define health, disease, degree of disease, or other conditions and exposures. Supervised learning (e.g., machine learning) is used to transform multivariate data from metabolite profiles into patterns that are biologically relevant (15–18). The general idea behind multivariate methods is to find, if possible, distinct metabolite profiles most strongly associated with the studied phenomenon (19). Once key metabolites have been identified, metabolic network analysis can generate hypotheses for a particular condition(s) (16). Other metabolomics analysis approaches include standard methods such as parametric/non-parametric univariate tests and ANOVA and more sophisticated methodologies. The use of multiple complementary statistical methods allows the investigator to extract the most important information from a single experiment (19). Figure 2 shows the typical workflow for metabolomics experiments and analysis.

## Methods

**Figure 3:**
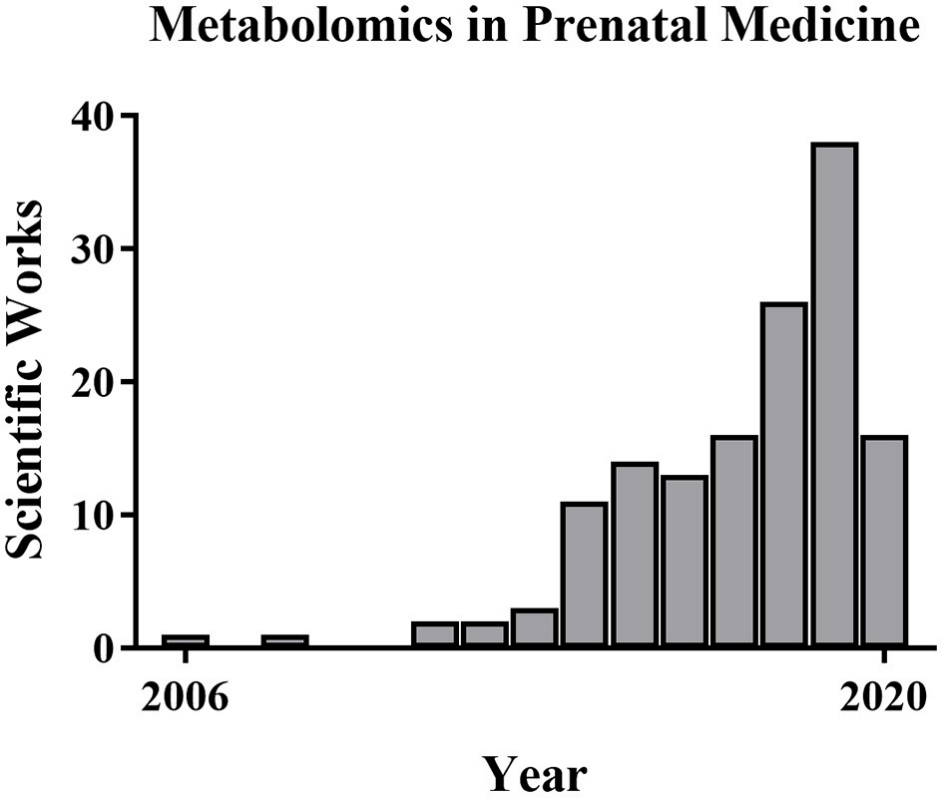
Metabolomics publications in prenatal medicine. The number of metabolomics publications in prenatal medicine is low but increased from 2006 to 2019 based on PubMed searches.

For this review of perinatal metabolomics, we conducted a comprehensive search of PubMed and Google Scholar throughout January 2020 using the following key terms: metabolomics, nuclear magnetic resonance, mass spectrometry, and metabolic profiling. We combined these with the following clinical terms: normal pregnancy, prenatal diagnosis, gestational disorders, chromosomal disorders, pre-eclampsia, fetal growth restriction, pre-term labor, and congenital anatomic defects. We identified additional articles by searching the reference lists of the identified studies. We considered peer-reviewed journal articles that appropriately described their methods and included only unique subjects, data, or analyses. We took into consideration the scientific papers issued between the years 2000–2020, thus observing that the larger number of them were mainly published in the last 10 years. Only papers dealing with the above-mentioned topics were included, those on other pathological conditions were omitted. Systematic reviews and meta-analyses were also excluded from the review together with studies focused on non-human subjects or studies published only in conference proceedings. Of 124 studies identified by the initial search, about 60 studies met the inclusion criteria. Table 2 reports the summary of each study. In particular, the data points taken from each study included the first author, year of publication, analytical technique, sample type and size, type of biomaterial examined, and results. Despite the fact that there have been a good number of pregnancy-related metabolite and metabolomics studies, the use of metabolomics specifically in prenatal diagnosis and pregnancy prediction has been more limited (63–65) although the number of pregnancy metabolomics studies is increasing (Figure 3). Next, we will review recent metabolomics publications for prenatal diagnosis and specific pregnancy pathologies.

**Table 2:** Key data points extracted from the cited studies.

	**Authors**	**Year**	**Pregnancy period**	**Analytical technique**	**Sample size**	**Biomaterial**	**Result**
Normal pregnancy physiology	Jauniaux et al. (20)	2004	1st trimester	HPLC GC-MS	24 HC	CF AF MP	CF vs. plasma: ↓ GSH, DHA, ↑α-tocopherol, ↓γ-tocopherol, ≈ Ascorbic acid, Uric acid CF vs. AF: ↑α-tocopherol, Ascorbic acid, Uric acid, ≈ DHA
	Heazell et al. (21)	2008	1st trimester	GC-TOF-MS	11 HC	P	With 1% O_2:_ ↑ 2-deoxyribose, erythritol, hexadecanoic acid
	Jauniaux et al. (22)	2005	1st trimester	HPLC	16 CF 12 AF 7 IF 17 MS	CF AF IF MS	CF and AF vs. MS: ↑ Inositol, sorbitol, erythritol, ribitol, fructose; ↓ glucose and glycerol CF vs. AF: ↑ inositol, sorbitol, erythritol, ribitol, mannitol, galactose, galactosamine, and glucosamine IF vs. MS: ↑ inositol, sorbitol, mannitol
	Murgia et al. (23)	2019	1st trimester	^1^H-NMR GC-MS HPLC	13 HC 8 CD	CV	Positive correlation with the CRL: Myo-inositol, glutamine, citrate. inositol, glycerol, dehydroascorbic acid, and ribitol Negative correlation with the CRL: 1,5-anydro-D-Sorbitol, D-fructose, and D-mannose
Aneuploidy screening	Troisi et al. (24)	2017		GC-MS	220 HC 108 CD	MS	CA vs HC:↓ Elaidic, mannose, stearic, myristic, ↑ benzoic, linolenic, citric and glyceric acid, 2-hydroxy butyrate, phenylalanine, proline, alanine and 3-methyl histidine
	Bahado-Singh et al. (25)	2013	1st trimester	^1^H-NMR	60 HC 30 T21	MS	Trisomy 21 vs HC: ↑ 3-hydroxybutyrate, 3-hydroxyisovalerate, and 2-hydroxybutyrate
	Bahado-Singh et al. (26)	2013	1st trimester	^1^H-NMR	114 HC 30 T18 30 T21	MS	Trisomy 18 vs. HC: ↑ 2-hydroxybutyrate, glycerol Trisomy 18 vs. Trsisomy 21: ↑ TMA, ↓ threonine,creatine, and formate
	Pinto et al. (27)	2015	1st/2nd trimester	^1^H-NMR	74 HC 45 CD	MP	CD vs. HC, 1st trimester: ↑ketone bodies; ↓ glucose, pyruvate, citrate, HDL, proline, methanol CD vs. HC, 2nd trimester: urea, creatinine, acetate, LDL, VLDL
	Diaz et al. (28)	2013	2nd trimester	^1^H-NMR	34 HC 33 CD 13 T21	U	Trisomy 21 vs. others CD: ↓ Glucose, N-methyl-2-pyridone-5-carboxamide
	Trivedi and Iles (29)	2015	1st/2nd trimester	ZIC-HILIC-IT-TOF RPLC-IT-TOF	93 HC 23 T21	U	Trisomy 21 vs. HC: ↑ Dihydrouracil, ↓ Progesterone
	Murgia et al. (23)	2019	1st trimester	^1^H-NMR GC-MS HPLC	13 HC 8 CD	CV	CD *vs* HC: ↑ Lactate, asparagine, branched-chain aminoacids, D-sorbitol, 1,5-anydro-D-sorbitol, D-fructose, dehydroascorbic acid, and glucose, ↓ myo-inositol, glycerol, fumarate, betaine, and acetate, cholesterol, pyruvic acid, palmitic acid, inositol, homoserine, stearic acid, GSH and GSSG
Pre-eclampsia	Dunn et al. (30)	2009	1st trimester	UPLC–MS	6 HC 6 PE	Explanted CV 1% O_2_ or 6% O_2_	PE 1% O2 vs. HC: ↑ Progesterone, Glycerol, Valinol or choline, Diglyceride. Alteration in glutamate and glutamine, tryptophan metabolism and leukotriene or prostaglandin metabolism
	Austdal et al. (31)	2014	2nd trimester	^1^H-NMR	10 HC preg. 10 HC not preg. 10 PE	U MS	Urine PE vs. HC pregnant: ↑ choline, ↓ glycine, p-cresol sulfate and hippurate Serum PE vs. HC pregnant: ↑ lipids, VLDL/LDL, histidine, glycerol
	Zhou et al. (32)	2017	Delivery	GC-MS	11 HC 11 PE	Placental mitochondria	PE vs. HC: ↓ ATP, citraconate and caprylate; ↑ arachidonate, bihomo-γ-linoleate, and γ-linoleate, docosapentaenoate, myristate in PE
	Bahado-Singh et al. (33)	2017	1st trimester	^1^H-NMR	55 HC 29 PE	MS	PE vs. HC: alteration in Branch chain amino acids
	Bahado-Singh et al. (34)	2015	1st trimester	^1^H-NMR	108 HC 50 PE	MS	PE vs. HC: ↑ 2-hydroxybutyrate, 3-hydroxyisovalerate, citrate, ↓ arginine, acetone, glycerol
	Bahado-Singh et al. (35)	2017	1st trimester 3rd trimester	^1^H-NMR LC-MS MALDI-TOF	35 PE 63 HC	MS	1st trimester: putrescine, urea and carnitine, TNF-α, RPL41, ATP5E, TBP 3rd trimester: methylhistidine, serotonin, citrate, hexose and propylene glycol, HLA-DR B1, GTP binding protein-3
	Koster et al. (36)	2015	1st trimester	UPLC-MS/MS	500 HC 68 early PE 99 late PE	MS	Early PE: combination of MC, MAP, PAPPA, PLGF, taurine, stearoylcarnitine Late PE: combination of MC, MAP, PAPPA, PLGF, stearoylcarnitine
	Kuc et al. (37)	2014	1st trimester	UPLC-MS/MS	500 HC 68 Early PE 99 Late PE	MS	Early PE vs. HC: ↓ taurine and asparagine Late PE vs. HC: ↓ glycylglycine
Fetal	Bernard et al. (38)	2017	2nd trimester 3rd trimester Post-natal	GC	1,171 Preg	MS	Linoleic acid positively associated with birthweight, BMI, head circumference, neonatal abdominal adipose tissue volume High DHA levels were associated with greater length/height
	Visentin et al. (39)	2017	3rd trimester	GC-MS	12 AGA 12 IUGR 10 SGA	MP FUVP	SGA vs. IUGR: ↑ C6:0 (in maternal plasma) SGA vs. AGA: ↑ C8:0, C10:0, and C12:0 (in maternal plasma) No statistical differences between AGA and IUGR MCFAs fetal to maternal ratio is >1 for IUGR group MCFAs fetal to maternal ratio is <1 for SGA and AGA
	Clinton et al. (40)	2020	1st trimester 2nd trimester	GC-MS	30 HC 30 FGR	U	1st trimester FGR *vs* HC: ↑ acetoacetate, 2-methylglutaric acid, benzoic acid 2nd trimester FGR *vs* HC: ↑ 1,2-propanediol, benzoic acid Increased level of cholesterol from 1^st^ trimester FGR to 2^nd^ trimester FGR
	Dessì et al. (41)	2014	Post-natal	^1^H-NMR	17 AGA 12 IUGR 9 LGA	U	IUGR vs. AGA: ↑ Myo-inositol, creatinine, creatine, citrate, betaine/TMAO glycine; ↓ urea, aromatic coumpounds, branched chain amicoacids LGA vs. AGA: ↑Myo-inositol, creatinine, aminoacids; ↓ urea, formate, citrate IUGR vs. LGA: ↑ Myo-inositol, creatinine, creatine, citrate, betaine/TMAO, glycine, acetate; ↓ urea, aromatic coumpounds
	Dessì et al. (42)	2011	Post-natal	^1^H-NMR	30 HC 26 IUGR	U	IUGR vs. HC: ↑ Myo-inositol, creatinine
	Favretto et al. (43)	2012	Post-natal	LC-MS	22 IUGR 21 AGA	FUVP	IUGR vs. AGA: ↑ Phenylalanine, tryptophan, and glutamate
	Sanz-Cortés et al. (44)	2013	Post-natal	^1^H-NMR	23 Early IUGR 23 AGA 56 Late IUGR 56 AGAs	FUVP	Early and late IUGR vs. HC: ↑ Unsaturated lipids and VLDL levels; ↓ phenylalanine, tyrosine, choline Early IUGR vs. HC: ↓ glucose; ↑ acetone, glutamine and creatine Late IUGR vs. HC: ↓ Valine, leucine
	Liu et al. (45)	2016	Post-natal	LC-MS	60 IUGR 60 AGA	Heel-stick blood	Newborns of different weight percentages: alteration in alanine, homocysteine, methionine, ornithine, serine, tyrosine, isovaleryl carnitine, and eicosenoyl carnitine IUGR vs. AGA: ↓ Alanine, homocysteine, methionine, ornithine, serine, and tyrosine Pre-term vs. full-term IUGR: ↑ homocysteine, heptanoyl carnitine decanoyl carnitine, methylmalonyl carnitine, glutaryl carnitine, sebacoyl carnitine, hydroxyacetyl carnitine, and hydroxyhexadecenyl carnitine
	Porter et al. (46)	2020	3rd trimester	LC-MS GC-MS	14 Low EFW 9 Normal UmA 5 Abnormal UmA 10 Normal UtA 3 Abnormal UtA	MP	Abnormal UmA vs. normal UmA: ↑ ornithine Abnormal UtA vs. normal UtA: ↓ dimethylglycine, isoleucine, methionine, phenylalanine, 1-methylhistidine
	Bahado-Singh et al. (47)	2020	Post-natal	^1^H-NMR DI-LC-MS/MS	30 HC 19 FGR	P	Combination of 3-hydroxybutyrate, glycine and PCaa C42:0 for FGR detection
	Sulek et al. (48)	2014	2nd trimester	GC-MS	30 Mother of SGA 42 Mother of HC	Hair	Combination of lactate, levulinate, 2-methyloctadecanate, tyrosine, and margarate
Pre-term labor and delivery	Caboni et al. (49)	2014	Term of gestation	GC-MS^1^H-NMR	59 Preg	U	Alanine, glycine, acetone, 3-hydroxybutiyric acid, 2,3,4-trihydroxybutyric acid and succinic acid characterize the late phase of labor
	Baraldi et al. (50)	2016	3rd trimester	UPLC-MS	13 Pre-term 11 Term	AF	PTD vs. TD: ↑ 3-methoxybenzenepropanoic acid, 4-hydroxy nonenal alkyne, muconic dialdehyde. ↓ phosphatidylcholine
	Graça et al. (51)	2010	2nd trimester	^1^H-NMR	27 Pre-term 82 Term	AF	Alanine, allantoin, citrate, and myoinositol
	Menon et al. (52)	2014	3rd trimester	GC-MS LC-MS	25 Pre-term 25 Term	AF	PTD vs. TD: Changes in Histidine metabolites (cis-urocanate, trans-urocanate, 1-methylimidazoleacetate) ↑ 4-acetamidophenol, 2-methoxyacetaminophen sulfate, 3-(cysteine-S-yl) acetaminophen, 3-(N-acetyl-L-cystein-S-yl) acetaminophen, p-acetamidophenylglucuronide, progesterone, bile acids; ↓ squalene, lathosterol, cortisolo, cortisone, metabolites related to caffeine, LCFAs, EFA, arachidonate, mead acid
	Romero et al. (53)	2010	2nd trimester	GC-MS LC-MS	52 Pre-term without IAI 60 Pre-term with IAI 56 Term	AF	Pre-term without IAI: ↓ carbohydrates and amino acids Pre-term with IAI:↓ carbohydrates; ↑ amino acids, Term:↑ mannose, galactose, fructose; ↓ alanine, glutamine, glutamic acid
	Virgiliou et al. (54)	2017	2nd trimester	UHPLC–MS	35 Pre-term 35 Term	AF MS	Pre-term (amniotic fluid): ↓ pyruvic acid, inositol, glutamine; ↓ glutamate Pre-term (serum): ↑ unsaturated lipids, pyroglutamic acid; ↓ hypoxanthine, tryptophan
	Lizewska et al. (55)	2018	Post-natal	LC-MS	57 Pre-term 49 Threatened pre-term labor 25 Term	MP	Threatened pre-term *vs* Pre-term and Term: ↓antiinflammatory omega 3, proinflammatory omega 6 fatty acids Pre-term *vs* Threatened pre-term: ↑ DHA
	Tea et al. (56)	2012	Post-natal	^1^H-NMR	35 VLBW 35 Term	FUVP MP	Fetal umbilical vein plasma *vs* Maternal plasma: ↑ amino acids, glucose, and albumin-lysyl; ↓ LDL, VLDL, lipid VLBW vs term: ↓ acetate; ↑ lipids, pyruvate, glutamine, valine, threonine
Congenital anatomic defects	Groenen et al. (57)	2004	2nd trimester 3rd trimester	^1^H-NMR	14 Spina bifida 18 HC	AF	Spina bifida *vs* HC: ↑ succinate, glutamine; ↓ creatine, creatinine
	Bock (58)	1994	2nd trimester 3rd trimester	^1^H-NMR	70 Preg	AF	PE: ↑ Choline, succinate, acetate Spina bifida: ↑ Lactate, glutamate, acetate
	Clifton et al. (59)	2006	2nd trimester 3rd trimester	^1^H-NMR	3 Preg	AF	3rd trimester vs. 2nd trimester: ↑ choline
	Pearce et al. (60)	1993	2nd trimester 3rd trimester	^31^P NMR	16 Preg	AF	Disaturated phosphatidylcholines positively correlates with the gestational age and fetal maturation
	Graça et al. (61)	2007	2nd trimester	^1^H-NMR	16 HC Preg	AF	Methodological article
	Graça et al. (62)	2009	2nd trimester	^1^H-NMR	51 HC 12 FM	AF	Fetal malformation vs HC: ↓ leucine, valine, ethanol, alanine, proline, glutamate, glucose; ↑ methionine, succinate, glutamine, citrate, glycine
	Bahado-Singh et al. (63)	2014	1st trimester	^1^H-NMR LC-MS	27 CD 59 HC	MS	CD vs. HC: ↓ C3-OH, C5-OH(C3-DC-M), C14:1, and SM C22:3, alteration in acetone, ethanol, acetate, and pyruvate levels
Single gene disorders	Monni et al. (64)	2019	1st trimester	GC-MS	27 HC 7 β-thal het 7 β-thal hom	CV	Homozygous vs HC and heterozygous: ↑ Glutamic acid, glycerol-1-phosphate, malic acid, arachidonic acid, glucose, and ribose; ↓ docosatetranoic acid, palmitoleic acid

*HC, healthy controls; CF, coelomic fluid; AF, amniotic fluid; IF, intervillous fluid; MS, maternal serum; MP, maternal plasma; U, urine; CV, chorionic villi; FUVP, Fetal umbilical vein plasma; P, placenta; CD, chromosomal disorders; T21, trisomy 21; T18, trisomy 18; T13, trisomy 13; PE, pre-eclampsia; FM, fetal malformation; Preg, pregnancies; MAP, Mean arterial pressure; TNF-α, Tumor necrosis factor-alpha; RPL41, 60S ribosomal protein L41; ATP5E: ATP synthase subunit epsilon; TBP, TATA box binding protein - associated factor; EFW, estimated fetal weight; UmA, umbilical artery; UtA, uterine artery; IUGR, intrauterine growth restriction; AGA, adequate-for-gestational-age; SGA, small-for-gestational-age; MCFAs, Medium chain fatty acids; PTD, pre-term delivery; TD, term delivery; LCFAs, long-chain fatty acids; EFA, essential fatty acids. IAI, intra-amniotic infection/inflammation; DHA, docosahexaenoic acid; HDL, High density lipoprotein; LDL, low-density lipoprotein; VLDL, very low-density lipoprotein*.

### Prenatal Diagnosis

Contemporary fetal assessment uses both non-invasive and invasive methods. Non-invasive methods include maternal factors and history, fetal ultrasound imaging, and maternal serum analyte or cell-free fetal DNA (cfDNA) screening. Maternal blood analyte tests, such as the first trimester screen (66), sequential screen, and quadruple test (67), and maternal serum cfDNA screening carry no procedural risk for the pregnancy, but these only assess risk and cannot diagnose. In contrast, invasive diagnostic tests require fetal samples of the placenta (chorionic villus sampling) or skin cells (amniocentesis) but provide definitive results. Invasive testing incurs a small chance of procedural complications, including a 0.1–0.2% risk of fetal loss (68). The earliest possible diagnosis of fetal aneuploidy and other congenital defects is highly desired because it affords more time for decision making and reduces risks of pregnancy interruption if that is what the couple chooses.

In first trimester non-invasive screening, we estimate risk from a combination of maternal factors (e.g., age, weight, and medical considerations, such as diabetes), fetal factors (e.g., nuchal translucency [NT], nasal bone, and fetal heart rate), and feto-placental factors in maternal blood (ß-hCG and PAPP-A). The combined first trimester screen detects trisomies 21, 18, and 13 with 90, 97, and 92% sensitivity but with a set positive rate of 5% (69). The second trimester quadruple test assesses α-fetoprotein, ß-hCG, estriol, and inhibin-A and detects trisomy 21 with ~80% sensitivity and a false positive rate of 5% (67). The newest non-invasive screening test is maternal serum cfDNA which exhibits very high sensitivity and specificity for trisomies 21, 18, and 13 by examining free chromosomal DNA fragments in maternal blood that are released by normal placental apoptosis (70). Although screening tests are all helpful for alerting us about fetuses at higher risk for aneuploidy, their positive predictive value varies dramatically depending on maternal age-related risk. Many women decline invasive testing despite the minimal risk and high diagnostic accuracy of CVS or amniocentesis. Therefore, developing better non-invasive tests that cover a broader range of disorders and have lower false-positive rates is a critical need in the field (71). Omics approaches could be a reasonable solution.

**Figure 4:**
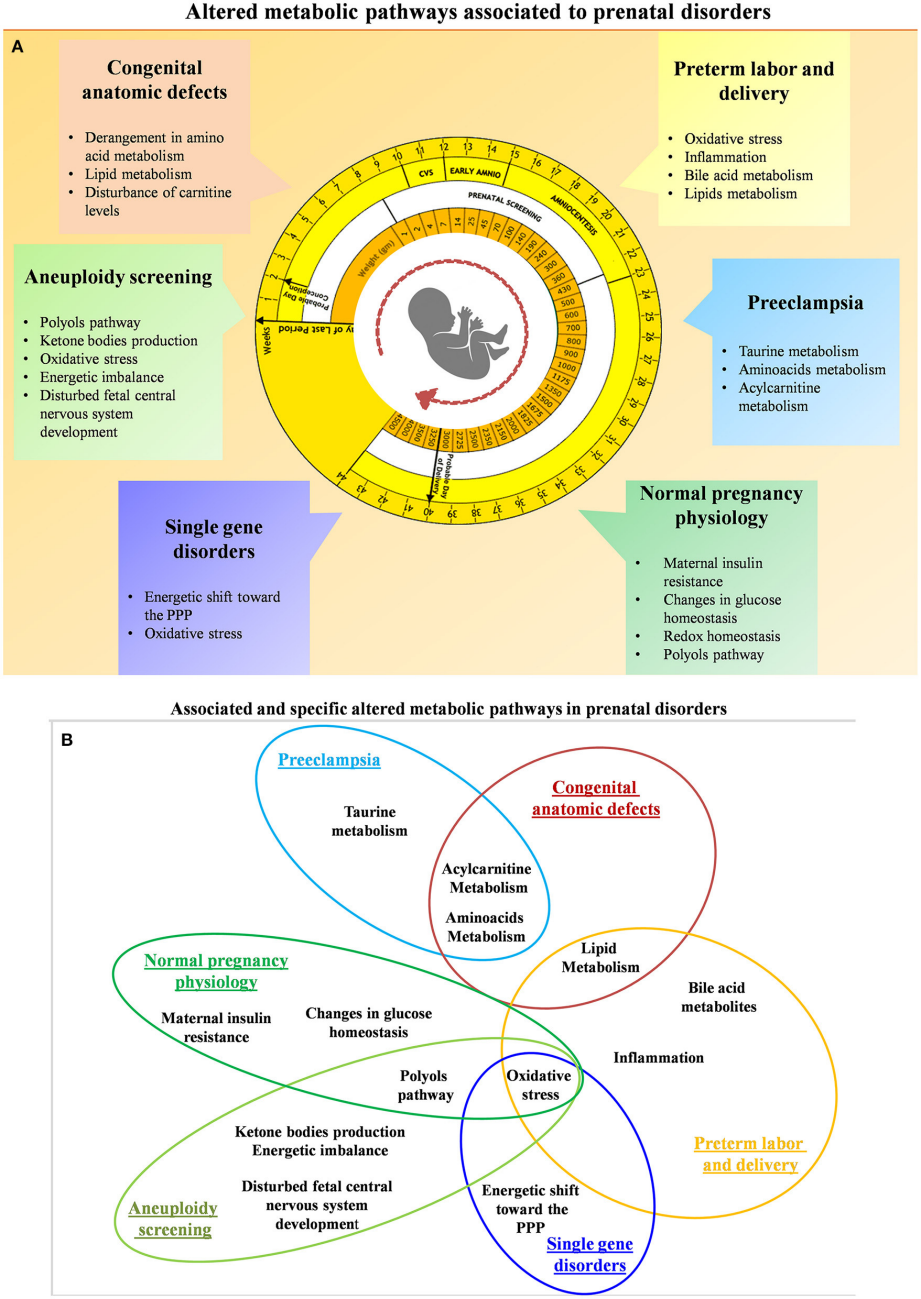
**(A)** Summary of the altered metabolic pathways associated with prenatal disorders. **(B)** Associated and specific altered metabolic pathways in prenatal disorders.

High-dimensional biology techniques (i.e., multi-omics) that combine genomic, transcriptomic, proteomic, and metabolomic approaches have been applied in both normal and complicated pregnancies (72–86). Because metabolomics provides a final readout of cellular physiology, it may be the best single omics approach to characterize normal vs. pathologic pregnancies. Examining different maternal-fetal compartments at different stages of pregnancy and clearly characterizing changes throughout gestation is required in order to determine the best tests and timing for particular clinical samples. Ideally, materia for non-invasive prenatal metabolomics would be obtained from maternal blood (plasma or serum) (87), urine (88), or cervico-vaginal secretions (89). Invasive sampling can also be used to obtain amniotic fluid (90), placental biopsy (23), and cord blood (43). Samples from invasive approaches might be useful to characterize the relationship between fetal and maternal metabolites or eventually might be used for confirmatory testing as it is today. Thorough metabolomic characterization of pregnancy across gestation might suggest new diagnostic or treatment options. So far, gestational metabolomics has been used to characterize normal physiologic changes of pregnancy as well as common complications including fetal aneuploidy, pre-eclampsia, pre-term birth, fetal growth restriction (FGR), pre-term parturition, congenital fetal anomalies, and single-gene disorders. A summary of the main investigated perinatal issues and the specific identified metabolic pattern is represented in Figures 4A,B. Below, we review recent work in each of these areas.

### Normal Pregnancy Physiology

Pregnancy requires a wide range of adaptive physiologic changes throughout gestation in maternal, fetal, and placental function. For example, circulating maternal metabolic products such as triglycerides, cholesterol, free fatty acids, and phospholipids change dramatically during pregnancy (91) to satisfy fetal energy and catabolic needs *in utero* and the production of an adequate maternal milk supply post-partum (92). Maternal insulin resistance also increases significantly with gestational age in normal pregnancy to ensure adequate transfer of glucose to the fetus (93). Maternal inositols increase with gestational age, positively correlating with fetal crown-rump length. They are correlated with insulin sensitivity and could be mechanistically linked to glucose homeostasis (91).

The placenta plays a key role in regulating the metabolic milieu of pregnancy. Several studies have described an altered placental metabolic profile, due to environmental factors. For example, the oxygen tension during placental explant culture *in vitro* can dramatically alter metabolic signatures. The human placenta is adapted for an initial hypoxic environment (20) then switches metabolism to accommodate increased oxygenation in the second trimester when extensive spiral artery remodeling occurs (94). The intervillous oxygen tension increases from 2–3% at 8 weeks to 8.5% by 12 weeks (95) of gestation with concurrent increased placental oxidative stress. Upregulation of placental antioxidant factors maintains redox homeostasis (96), and the metabolic profile of normal placental villus explants varies with oxygen culture conditions (21). Changes in hexadecanoic acid, erythritol, and 2-deoxyribose are particularly prominent. Moreover, placental cholesterol levels were found to be increased in correlation with the CRL. The higher concentration of cholesterol may be the result of increased levels of progestational hormones. In fact, maternal blood cholesterol represents the precursor of both progesterone and estrogen (23). Other gestational age-related metabolic changes (22, 23) include placental polyol pathways which are very active in the first trimester. One hypothesis regarding elevated polyols in early pregnancy is that they are an early carbohydrate source for the placenta and embryo. Additionally, the polyol pathway may facilitate the re-oxidation of pyridine nucleotides under low oxygen conditions, helping regulate intracellular pH during periods of robust glycolysis (23). These are just a few examples of the way in which metabolomics has been used to characterize the normal physiologic changes of pregnancy.

### Aneuploidy Screening

Chromosomal abnormalities are the most frequent fetal problem diagnosed in the first trimester. Trisomy 21 (T21; Down syndrome), trisomy 18 (T18; Edwards syndrome), trisomy 13 (T13; Patau syndrome), and abnormal sex chromosomes (e.g., XO, Turner syndrome, or Monosomy X) are the most commonly screened chromosome errors. Standard routine prenatal care includes screening for these common aneuploidies (97). Although current screening tests (see above) successfully identify fetal aneuploidy in early pregnancy, the search for additional genetic biomarkers for a wider range of aneuploidies and improved sensitivity and specificity is an important goal in the science of prenatal diagnosis (24).

Several studies have examined the utility of metabolic screening for aneuploidy. For example, Bahado-Singh et al. used NMR to analyze first trimester maternal serum from T21 and control pregnancies (25). They found 11 metabolites differed between groups, including the novel biomarkers 3-hydroxybutyrate and 2-hydroxybutyrate. The first is an indispensable energy source for extrahepatic tissues such as the brain, involved in growth and myelination; the second is involved in oxidative stress defense (25). Bahado-Singh similarly studied first trimester maternal serum from normal and T18 pregnancies (26) and found that glycerol and 2-hydroxybutyrate best identified T18 fetuses. Combining discriminatory metabolites with clinical and demographic parameters, they detected T18 with 90% sensitivity and 100% specificity using the delta nuchal translucency (98) and 2-hydroxybutryate levels together. Several other metabolites appear to distinguish between T18 and T21 fetuses including threonine, trimethylamine, creatine, and formate (26). Troisi et al. published similar findings for pregnancies affected by any trisomy (combined group of T21, T18, and T13) vs. normal controls (24). A specific pattern of metabolites was altered in trisomic compared to normal fetuses, suggesting a metabolic environment of elevated oxidative stress and disturbed fetal central nervous system development. Yet another group found associations between T21 and abnormal high-density lipoprotein (HDL), methanol, and proline in the first trimester and abnormal creatinine, acetate, HDL, and low-density lipoprotein (LDL) in the second trimester (27). Overall, these studies suggest increased oxidative stress in aneuploid pregnancies and switched fuel metabolism resulting in β-oxidation and ketone body production. Aneuploid fetuses appear to utilize glucose, pyruvate, and citrate less as energy sources compared with normal pregnancies.

In addition to maternal plasma and serum, some metabolomics studies of aneuploidy have evaluated maternal urine specimens. Maternal urine metabolic signatures for chromosomal disorders generally showed altered 3-hydroxybutyrate, 2-ketoglutarate, and 1-methylhistidine. Urine metabolites specifically associated with T21 included N-methyl-2-pyridone-5-carboxamide (28). Trivedi and Iles clarified the altered cellular metabolism with T21, suggesting that metabolic profiles may improve detection of both aneuploidy and inborn errors of metabolism (29). Comparing the mass spectrometry urine metabolome of women with aneuploid or normal fetuses revealed altered levels of progesterone and dihydrouracil (29).

Our own laboratory has characterized metabolic networks from the first trimester placenta obtained via transabdominal chorionic villus sampling (TA-CVS) (23). CVS placental biopsies are ideal for metabolic analyses as they are obtained from the undisturbed placenta *in situ* rather than after delivery, miscarriage, or termination. We compared normal and aneuploid fetuses (T21, T18, T13) using NMR, GC-MS, and HPLC and found critical differences in energy metabolism and polyol pathways. The aneuploid placenta demonstrates excessive polyol pathway activation, decreased glutathione levels, and increased dehydroascorbate. Additionally, thorough characterization of the placental metabolome may significantly improve our ability to interpret changes in the maternal metabolic profile caused by fetal or placental dysfunction.

### Pre-eclampsia

Pre-eclampsia (PE) is a gestational hypertensive syndrome that complicates 2–8% of pregnancies worldwide (99) and is a major cause of maternal/fetal morbidity and mortality. The current definition of PE, according to the International Society for the Study of Hypertension in Pregnancy (ISSHP) (100) and the American College of Obstetricians and Gynecologists (ACOG) (101), is a new onset of hypertension (blood pressure ≥140 mmHg systolic or ≥90 mmHg diastolic) at ≥20 weeks of gestation and proteinuria (≥300 mg/24 h or protein-to-creatinine ratio >30 mg/mmol or ≥2+ on dipstick testing) or cases without proteinuria but with severe range blood pressure (>160/110) or evidence of organ dysfunction (e.g., hematologic, renal, hepatic, and neurologic). This new definition of PE resulted in an increase in pregnancies diagnosed with PE but generally milder disease (102).

Although the exact cause has not been identified, PE is thought to be due to the interaction of maladaptive trophoblast-derived signaling factors [e.g., soluble FMS-like tyrosine kinase-1 (sFlt-1) and placental growth factor (PlGF)] (103, 104) with susceptible maternal physiology. One theory is that PE is triggered by reduced uterine perfusion leading to placental oxidative stress and apoptotic release of pro-pre-eclampsia signals. Despite decades of PE research, the only available treatment remains delivery (i.e., removal of the placenta), and the only marginally helpful prevention comes from maternal risk reduction and possibly low-dose aspirin prophylaxis (105). Combined screening using maternal factors, mean arterial pressure, uterine artery, Pulsatility Index (PI), and maternal serum PlGF has been proposed to predict about 90% of early PE (Early-PE; <34 weeks), 75% of late pre-term PE (Late Pre-term-PE; 34 to <37 weeks) and 45% of full-term PE (Term-PE; ≥37 weeks) (106). Identifying and integrating new biomarkers to predict PE prior to symptoms could facilitate diagnosis and prevention. Given the heterogeneous presentation of gestational hypertensive disorders, it is unlikely that only one or several biomarkers will predict all types of PE (e.g., gestational hypertension, PE, PE with severe features, HELLP, and eclampsia). Therefore, broad network analysis using metabolomics may be the most appropriate approach to solving this important recalcitrant clinical problem.

Several groups have examined differences in maternal metabolomics in normal and PE pregnancies. *In vitro* studies of villous trophoblast explants using LC-MS identified differences in culture media metabolites for normal and PE patients (30). Furthermore, the metabolic profile changes in response to different oxygen conditions varied between normal and PE placenta, suggesting not only baseline differences in PE metabolism but persistent functional differences in the response environmental stressors. Studies have not determined whether the changes in hypoxia responses *in vitro* preceded PE or were caused by it (30). *In vivo* changes due to PE have been studied in maternal urine and serum (31). Women with PE have increased urine markers of oxidative stress and renal or liver dysfunction as well as altered serum metabolites. In particular, higher total lipid content and lipoprotein levels were observed with PE (31). One study used metabolomics and Western blotting to examine placental mitochondrial funding during severe PE. Isolated placental mitochondria from severe preeclamptics showed reduced ATP levels, higher fatty acid levels, and decreased fatty acid catabolism (32).

Some of the most intriguing prenatal diagnostic studies in PE research have retrospectively assessed early metabolic changes from normal control pregnancies vs. PE cases. For instance, Bahado-Singh et al. investigated serum metabolic profiles in patients with early and late-onset PE compared with healthy controls using specific metabolic panels (33, 34). The metabolites 3-hydroxyisovalerate, arginine, and glycerol were particularly increased in Early-PE, especially when combined with uterine artery PI. An approach using metabolic, proteomic, and ultrasound parameters reached over 90% sensitivity and nearly 90% specificity for predicting PE. An important milestone in the PE study has been filed when metabolomic was combined with proteomic approaches (35) and clinical maternal features to enhance omics diagnosis from early pregnancy maternal blood samples. Significant changes in G-protein-coupled receptors, signal transduction serotonin, and glycosaminoglycan metabolisms emerged as the final result.

Similarly, Koster et al. constructed a prediction model using maternal clinical factors (e.g., maternal age, history of pre-eclampsia, other risk factors) for baseline risk and then adding protein and metabolite measures (36). More in detail, acylcarnitines of first-trimester maternal serum from women with and without PE were analyzed and correlated with the clinical factors. Interestingly, the correlation between markers selected for prediction modeling (hexanoylcarnitine, octanoylcarnitine, decenoylcarnitine, and decanoylcarnitine) showed an *R* > 0.8, suggesting a potential role of this class of compounds. That approach reached ~70% sensitivity and 90% specificity for Early-PE. For Late Pre-term-PE, the sensitivity and specificity were only ~30 and 90%. Moreover, these findings suggested the stearoylcarnitine as a biomarker for both EO- and LO-PE. The concentration of this biomolecule improved the prediction power of the clinical factors normally used to evaluate the baseline risk.

An important critique of PE metabolomics studies (and metabolomics diagnostic studies in general) is the wide variability in reported significant metabolites by different groups. Indeed, some studies evaluating metabolites for PE prediction have found that clinical risk factors are better than metabolite levels and that metabolite assessment does not add predictive power (37). Nonetheless, further studies may identify consistent factors we might use to create accurate, sensitive, specific prediction algorithms for PE (35).

### Fetal Growth Restriction

Fetal growth restriction (FGR) is defined by a fetus that fails to meet expected growth for gestational age using estimated fetal weight below the 10th percentile. FGR occurs in 5–10% of all pregnancies (107) due to multiple causes and increases the risk of adverse perinatal outcomes. The primary challenge for FGR is that fetuses measuring below the 10th percentile for gestational age may be either constitutionally small for gestational age (SGA) or have pathologic growth restriction. Thus, there is no agreed-upon single gold standard for FGR diagnosis, and the possibility of developing metabolomics approaches to diagnose FGR pathology has generated significant interest.

Several metabolomics studies have highlighted abnormal lipid metabolism during pregnancies resulting in SGA newborns (38, 108). Interestingly, near delivery, the mother-to-newborn ratio of medium-chain fatty acids was decreased with FGR, suggesting increased energetic and structural metabolic demands of the infant (39). Differences in cholesterol synthesis were observed in FGR fetuses compared to normal fetuses (40). In particular cholesterol levels during pregnancy only increased slightly in FGR cases (2.48-fold change) while increasing substantially in normally grown controls (6.54-fold change). In neonatal urine, newborns from FGR pregnancies had increased myo-inositol, which correlated with downregulation of adipose free fatty acid release (41, 42).

Amino acid metabolism also appears to play a role in FGR, with several amino acids differing between the average for gestational age and FGR newborns (43, 44) especially ornithine (45). A role for ornithine in fetal growth was also identified in maternal serum from pregnancies affected by extreme FGR (<5th %ile) and abnormal umbilical artery Dopplers (46). Correlations between ornithine and umbilical blood flow may be related to angiogenesis via polyamine pathways. Placentae from FGR pregnancies show almost universally decreased metabolite levels (47), and generally reduced metabolite concentrations have also been detected in the maternal hair from FGR pregnancies (48). Recent mechanistic research suggests that there is a general disruption in fetal energy substrates and metabolism in FGR, but the unique metabolic adaptations of FGR vs. SGA have not been well-studied.

### Pre-term Labor and Delivery

About 10% of all deliveries occur pre-term at <37 weeks. Pre-term birth (PTB) and the consequences of prematurity are the greatest contributors to neonatal morbidity and mortality (109, 110). Prematurity also has important long-term consequences for lifelong health and disease, and survivors of prematurity have increased risk for chronic medical conditions such as cardiovascular disease, metabolic syndrome, stroke, dyslipidemia, and neurocognitive dysfunction (111–113). Idiopathic pre-term labor (PTL) and pre-term prelabor rupture of membranes (PPROM) account for about two-thirds of pre-term deliveries while indicated deliveries (e.g., pre-eclampsia, placental abruption) account for the other third (114). PTB is the common pathophysiologic endpoint of a wide range of causes, and decades of research have failed to develop effective treatments or even provide adequate screening algorithms (115). Omics-sciences could play a role in defining normal pre-term/term physiology and the pathophysiologic pre-term processes leading to early labor and delivery (116, 117). Some have suggested that all of the great obstetrical syndromes, including PTB, represent different manifestations of various placental dysfunction or maladaptation which might be characterized effectively using metabolic assessment.

Many PTB investigators have focussed on specific markers of inflammation. Inflammation signaling is frequently associated with pre-term labor/PTB (118, 119), but a single universal marker or even several markers may not be sufficient to characterize the diverse etiologies leading to the common final pathway of labor. Analysis of urine from pregnant women before and after labor onset with GC/MS and NMR found 18 unique metabolic changes associated with labor status (49). Analysis of amniotic fluid metabolites has been of particular interest, as the fetus and placenta may be a critical cog in the pregnancy clock (50–54). One study compared amniotic fluid and maternal serum, identifying energy metabolism factors-associated PTB in the fetal compartment (pyruvate, glutamate, and glutamine) that were distinct from maternal serum metabolites that discriminated PTB (54). Another study used amniotic fluid metabolic profiling to compare women with pre-term contractions at risk for spontaneous PTB with or without intra-amniotic infection. Altered amniotic fluid carbohydrates were associated with PTB regardless of infection, while increased amino acids were present only with PTB and intrauterine infection (53). Graca et al. found decreased amino acids, citrate, and myo-inositol and increased allantoin and hexose in amniotic fluid from second trimester PTB cases (51).

Metabolic fingerprinting of pre-term labor that goes on to PTB has been attempted (55). This is a critical challenge in the field, as the majority of PTL does not proceed to PTB. Several studies found increased fatty acids associated with PTB (53). PTB was also associated with decreased levels of acetate and increased lipids (56) and a few amino acids. Interestingly, there may be important differences in PTB metabolic signatures by race and ethnicity. Menon et al. evaluated amniotic fluid from African American women with early spontaneous PTB vs. term birth. Bile acids, steroids, and xanthines were altered and the authors found 8-fold increased pantothenol levels in women who delivered early (52). To achieve the full potential of metabolomics in PTB research, careful selection of comparison groups and rigorous deep phenotyping of both maternal and fetal characteristics is needed.

### Congenital Anatomic Defects

It is well-known that some congenital/structural defects lead to changes in perfusion, organ function, or other factors, which could change the fetal metabolic signature and perhaps be reflected in maternal serum. Thus, even though congenital structural defects are not metabolic issues *per se*, we may detect some anomalies through metabolic approaches. We might also exploit metabolomics to elucidate causative metabolic mechanisms. Although NMR of amniotic fluid has been used for decades to characterize neural tube defects (57) and fetal lung or kidney maturity (58–60), more recently NMR has been paired with MS to identify other malformations. Graça et al. evaluated second-trimester amniotic fluid from normal pregnancies and from fetuses affected by congenital anatomic anomalies (61, 62). Surprisingly, maternal and fetal characteristics (e.g., maternal age and fetal sex) had no effect on the metabolic profile of normal fetuses. However, in multivariate supervised analysis, specific changes in glucose, succinate, and some amino acids and proteins were clear (62). Overall, these changes suggested a shift to glycolysis, perhaps due to hypoxic stress. Thus, it is possible that metabolic assessment could be used to screen for anatomic anomalies in combination with detailed fetal ultrasound imaging, similar to maternal serum alpha-fetoprotein for neural tube and abdominal wall defects.

Metabolomics has also been used to specifically evaluate fetal congenital heart disease (CHD) (120, 121). A few studies report metabolic screening for both structural and functional fetal heart disorders. For example, one report described metabolic changes in first trimester maternal serum for chromosomally normal fetuses with structural heart disease, showing altered phosphatidyl-choline and sphingolipids (122). Whether these metabolic changes were related to cardiac energetic processes or other end-organ compensation for altered heart function (e.g., liver dysfunction) was not determined (122). This sort of finding suggests that metabolic characterization could be used as an additional tool for CHD screening.

### Single-Gene Disorders

Common single-gene disorders are routinely evaluated with parental carrier testing and increasingly with cfDNA sequencing techniques. It is possible that some single-gene disorders, even those not affecting biochemical pathways, could be detected or at least flagged for increased scrutiny using metabolomics. That would be particularly helpful for *de novo* mutations. In some cases, phenotypic or genetic heterogeneity can make prenatal diagnosis difficult (e.g., β-thalassemia, Noonan's syndrome). Nonetheless, Monni et al. found that pregnancies affected by β-thalassemia exhibit significant metabolic changes (123). Comparing the metabolic profiles of placental samples obtained by TA-CVS from normal fetuses and fetuses with homozygous or heterozygous β-thalassemia identified consistent alterations in all β-thalassemia cases. We have proposed a specific metabolic fingerprint for β-thalassemia that is associated with high fetal demand for ribose 5-phosphate (for nucleotide synthesis) and nicotinamide adenine dinucleotide phosphate (for redox maintenance). It appears that fetal oxidative stress can be an important and frequent marker for a wide range of abnormal conditions, and metabolic markers of oxidative stress are readily detected with metabolomics techniques. Other heterogenous single-gene disorders may have common metabolic phenotypes as well that might be best detected by the end products of biochemical production through the analysis and quantification of metabolomics pathways.

## Conclusions

Metabolomics is a novel and promising area of research in reproductive medicine. It can be placed in the field of precision medicine, aiming, in general, at developing personalized strategies to manage disease states by considering, at the same time, the patient's genetics, environment, lifestyle, and individual treatment responses. Considering the current prenatal screening methods, it is clear that genetics plays the most relevant role, but metabolomics can generate new insights into the biological and physio/pathological processes. In this perspective, metabolomics can offer the opportunity to find new therapeutic targets and a better understanding of pathological mechanisms.

Table 3 summarizes the advantages and limitations of metabolomics in prenatal medicine. Metabolite detection with high throughput systems coupled with advanced bioinformatics and network analysis holds promise for new prenatal biomarkers and therapeutic discoveries. This metabolomics approach can identify complex physiologic pathways that would not be detected by measuring single metabolites. Further metabolomic investigation of both normal and pathologic prenatal specimens may enhance our knowledge of pregnancy disorders and improve our ability to diagnose and treat fetal disease. Toward this goal, the implementation of large-scale metabolomics studies and secondary cohort validation will be needed. Indeed, despite the general success and the increasing number of publications in prenatal medicine as well, the impact of the metabolomics in the current clinical practice is still dim. Several aspects contribute to this statement: one of the main problems is the enormous variability (external stimuli not closely related to the disease conditions, such as diet, lifestyle in general, analytical and experimental conditions, and data analysis methods), which could influence the final result. Thus, the experimental design covers a fundamental significance and should be planned with extremely controlled conditions. In addition, based on our literature investigation, it has emerged that several clinical metabolomics research studies are affected by strong limitations (e.g., small size of the patient cohorts and incomplete patient's clinical data), and often studies with the same topics produce results that are poorly comparable. Standardization of the methods is mandatory to promote the translation of the metabolomics toward clinical practice. It is important to consider that unfortunately, validated findings have not been yet evidenced in the literature, probably by being metabolomics in prenatal medicine a pioneer new research field. For this reason, new works are still expected in this context. Clinicians who are interested in learning these new approaches and participating in such studies have much to offer the field. Hopefully, soon we will be able to offer our patients clinical metabolomics tools to more effectively characterize, diagnose, and develop treatments for a vast range of pregnancy conditions and fetal disorders.

**Table 3:** Advantage, limitations, and the future of metabolomics in prenatal medicine.

**Metabolomics in prenatal medicine**	
**Advantages**	**Limitations and future directions**
Evaluates several biomarkers in a single experiment	Possible over-interpretation of data
Rapid experimental turnaround and relatively low cost	High false discovery rates requiring expert analysis
Does not require a-priori hypotheses of specific metabolites	Proof of initial findings in cell line and animal models often lags initial reports
Can identify altered metabolic pathways from multiple metabolite analysis	Hypothesis generating approach, but cutoff values and normal ranges must be established for clinical studies
May permit earlier identification of fetal or pregnancy disorders	Collaboration is weak among clinicians, analytical chemists, and biotechnologists
Simultaneously analyze metabolome of several compartments (e.g., maternal, placental, fetal)	Simple, specific tests that do not use sophisticated equipment may need to be developed

## Author Contributions

FM and VC: conceptualization. FM, VC, and FD: methodology. LA and GM: resources. FM and AI: data curation. FM and KH: writing–original draft preparation. FM, KH, LA, and GM: writing–review & editing. GM: supervision. All authors contributed to the article and approved the submitted version.

## Conflict of Interest

The authors declare that the research was conducted in the absence of any commercial or financial relationships that could be construed as a potential conflict of interest.
